# Motor outcome and electrode location in deep brain stimulation in Parkinson's disease

**DOI:** 10.1002/brb3.1003

**Published:** 2018-05-30

**Authors:** Maija Koivu, Antti Huotarinen, Filip Scheperjans, Aki Laakso, Riku Kivisaari, Eero Pekkonen

**Affiliations:** ^1^ Department of Neurology Meilahti Hospital Helsinki University Hospital Helsinki Finland; ^2^ Department of Neurosurgery Töölö Hospital, Helsinki University Hospital Helsinki Finland

**Keywords:** deep brain stimulation, electrode position, outcome, Parkinson's disease, subthalamic nucleus

## Abstract

**Objectives:**

To evaluate the efficacy and adverse effects of subthalamic deep brain stimulation (STN‐DBS) in patients with advanced Parkinson's disease (PD) and the possible correlation between electrode location and clinical outcome.

**Methods:**

We retrospectively reviewed 87 PD‐related STN‐DBS operations at Helsinki University Hospital (HUH) from 2007 to 2014. The changes of Unified Parkinson's Disease Rating Scale (UPDRS) part III score, Hoehn & Yahr stage, antiparkinson medication, and adverse effects were studied. We estimated the active electrode location in three different coordinate systems: direct visual analysis of MRI correlated to brain atlas, location in relation to the nucleus borders and location in relation to the midcommisural point.

**Results:**

At 6 months after operation, both levodopa equivalent doses (LEDs; 35%, Wilcoxon signed‐rank test = 0.000) and UPDRS part III scores significantly decreased (38%, Wilcoxon signed‐rank test = 0.000). Four patients (5%) suffered from moderate DBS‐related dysarthria. The generator and electrodes had to be removed in one patient due to infection (1%). Electrode coordinates in the three coordinate systems correlated well with each other. On the left side, more ventral location of the active contact was associated with greater LED decrease.

**Conclusions:**

STN‐DBS improves motor function and enables the reduction in antiparkinson medication with an acceptable adverse effect profile. More ventral location of the active contact may allow stronger LED reduction. Further research on the correlation between contact location, clinical outcome, and LED reduction is warranted.

## 1 INTRODUCTION

Deep brain stimulation (DBS) has been demonstrated in randomized studies to be more effective than optimal medical treatment alone in Parkinson's disease (PD; Deuschl et al., 2006; Weaver et al., 2009). DBS alleviates motor symptoms and reduces levodopa‐induced dyskinesia (Okun et al., 2012; Schüpbach et al., 2013; Williams et al., 2010). This benefit has been reported to last over 5 years (Bronstein et al., 2011; Krack et al., 2003; Rodriquez‐Oroz, Moro, & Krack, 2012; Schüpbach et al., 2005).

Although available since 1995 in Finland, only few DBS studies have been published (Erola et al., 2005; Heikkinen et al., 2004). This retrospective study is the first review of DBS outcome in Finland on a larger scale. The objective of this study was to evaluate the outcome of DBS‐treated patients with advanced PD at Helsinki University Hospital (HUH) between 2007 and 2014.

We analyzed the changes of Unified Parkinson's Disease Rating Scale (UPDRS) part III score, Hoehn & Yahr stage (H&Y), the decrease in antiparkinsonian medication, and adverse effects. Furthermore, in this study, the possible correlation between the electrode location and clinical outcome was addressed. This issue has recently gained more attention in DBS‐related follow‐up studies (Garcia‐Garcia et al., 2016; Johnsen, Sunde, Mogensen, & ØStergaar, 2010; Nestor et al., 2014; Paek et al., 2011, 2013; Tsai et al., 2007; Welter et al., 2014).

## 2 MATERIALS AND METHODS

The medical history of 103 patients with PD who underwent STN‐DBS at HUH between 2007 and 2014 was reviewed. In 87 patients, all the necessary data and sufficient imaging were available, and the assessment of the active electrode contact could be made. The clinical inclusion criteria for DBS operation were idiopathic PD with suboptimal response to conventional medication, one or more of the following symptoms: daily ON/OFF fluctuations, severe dyskinesia or drug‐resistant rest tremor. Positive response to levodopa was required during challenge test. Contraindications were dementia, existing psychosis or severe depression, clinical or radiological suspicion of atypical parkinsonism and/or a significant brain atrophy or vascular changes seen in brain MRI.

To assess the clinical outcome of DBS, UPDRS part III (UPDRS‐III) scores at baseline in medication OFF state was chosen for evaluating the baseline severity of Parkinson's disease and at 6 months in medication OFF and DBS ON state to evaluate the efficacy of DBS stimulation. Secondary outcomes were changes of H&Y stage and dopaminergic medication calculated in levodopa equivalent dose, LED, as suggested by Thomlinson et al. (2010). LEDs were obtained at baseline and at 6 months. H&Y staging was derived from the medical records during the pre‐DBS screening and at 6 months. The stimulator settings (voltage, pulse width, polarity, and frequencies) at 6 months were used for analysis. Complications including intracerebral hemorrhage (ICH), infection and dysarthria were reviewed. An infection was defined as requiring treatment with antibiotics and/or revision.

The DBS operation including targeting and imaging was conducted according to common clinical practice and the choice of the operating surgeon. Each patient had a preoperative brain MRI without a frame before the operation day and a CT scan with an attached Leksell stereotactic frame on the morning of DBS operation. In most cases, the targeting was performed by two surgeons individually and the final coordinates were concluded by comparing the two coordinate sets. The DBS operation was performed under light sedation and local anesthesia. Intraoperatively, the accuracy of implantation was confirmed with X‐ray fluoroscopy in AP and lateral directions. Immediately after fluoroscopy, all four contacts were tested intraoperatively for side effects and clinical benefit while the patient was awake.

Analyses of electrode location were performed from MRI and CT scans acquired as part of clinical routine and no extraimaging was made for this study. Image analysis was performed with Agfa Healthcare N.V.'s Impax (version 6.5.5.1608, Belgium) and Brainlab iPlan (version 3.0.5, Germany) stereotactic software. Postoperative CT scans (on the 1. or 2. postoperative day) were reviewed for complications and to determine the amount of intracranial air. The amount of midbrain midline shift compared to skull midline was measured from preoperative and postoperative CT. Postoperative CT controls were fused to preoperative MRI scans using whole brain MRI and CT with the target area visually inspected for sufficiently accurate fusion, which was realigned in AC–PC orientation. Six months' DBS programming record entry was used to find the active electrical contact (defined as the negative electrode) while the implanted pulse generator, implantable pulse generator (IPG) was the positive. It was recorded whether the stimulation was bipolar or monopolar. If there were two active negative contacts, the target point was defined as the middle point between these two contacts. To define the MCP, the anterior and posterior commissure were identified preferentially from 1.5 T T2 image and the anatomical analysis of the target area was performed preferentially from 1.5 T susceptibility weighed images (SWI, SWAN; Vertinsky et al., 2009). In case of suboptimal image quality of these pre‐DBS images, we used previous 3 T MRI scans obtained during screening. The MRI images were reconstructed in axial, sagittal and coronal planes as defined by anterior and posterior commissure before electrode location analysis. Images of MRI and CT fusion and of method of analysis of active electrode location can be found in Supplementary Materials.

The location of the active electrode was determined in three coordinate systems: direct visual analysis of the MRI scans which was correlated by the researcher to the Mai atlas, location in relation to the NR borders (anterior, lateral and superior), similarly as described by others, and location in relation to MCP (Houshmand, Cummings, Chou, & Patil, 2014; Mai, Paxinos, & Voss, 2008; Rabie, Verhagen Metman, & Slavin, 2016; Slavin, Thulborn, Wess, & Nersesyan, 2006). The target coordinates were expressed in relation to midcommissural point (MCP) in anterior commissure–posterior commissure (AC–PC) coordinates and by a method based on direct MRI visualization of the subthalamic nucleus (STN) and/or the nucleus ruber (NR; Rabie et al., 2016).

The location was recorded in all three dimensions as *X*‐, *Y*‐, and *Z*‐coordinates representing mediolateral, antero‐posterior, and dorsoventral directions. The location of the median coordinates of electrodes was compared between patients with less than 30% and those with 30% or more reduction in LED between baseline and at 6 months.

Statistical analysis was performed with IBM SPSS Statistics (version 22.0.0, Armonk, NY, USA). Data are presented as median (interquartile range, IQR). Data analysis was carried out using Mann–Whitney *U*‐test when appropriate. Correlations were calculated with Spearman correlation. The study was approved by the HUH Medical Ethics Committee.

## 3 RESULTS

There were 103 PD‐related STN‐DBS operations in HUH between 2007 and 2014. For 87 patients, necessary data and sufficient imaging were available and the precise definition for active contacts could be made. Fifty‐three (61%) patients were men and 34 (40%) women. Median age was 61.0 (IQR 54.0–65) years. The median delay from PD diagnosis to DBS operation was 11.0 (IQR 8–15) years. At baseline, UPDRS‐III was 37.0 (IQR 31.0–48.0), H&Y 2.5 (IQR 2.0–3.0) and LED 1,117 mg (IQR 793–1, 451 mg). Medtronic Kinetra and PC pulse generators (IPG) were implanted, most of IPGs being PC. In 6 months follow‐up, no deaths were noted.

**Figure 1:**
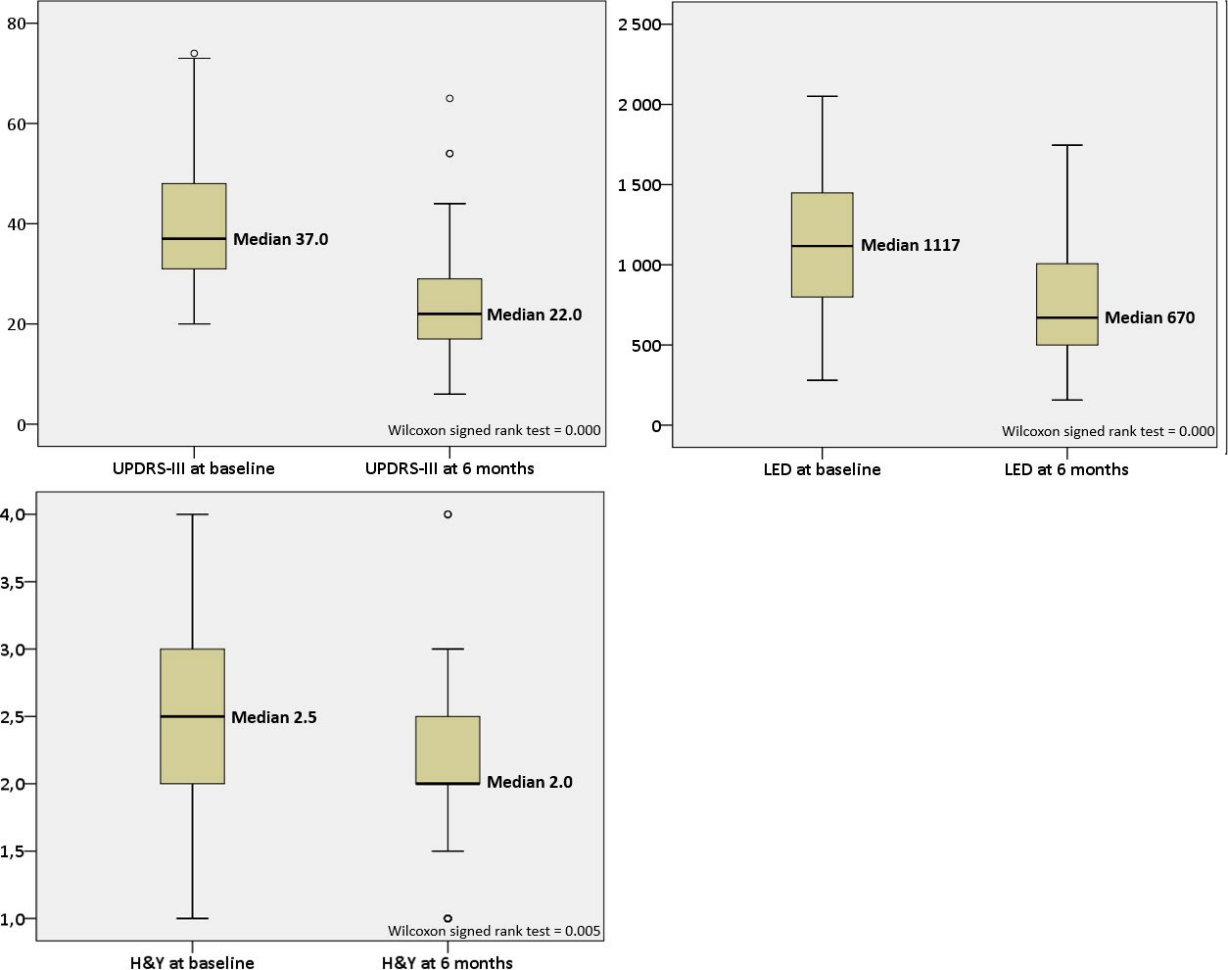
UPRDS III, LED and Hoehn & Yahr (H&Y) at baseline and at 6 months. Heavy line representing median, the size of the boxes interquartile ranges and whiskers minimum and maximum scores (range). Circles representing legitimate outliers. The active electrodes of the outliers did not statistically differ from other patients

Baseline and 6‐month UPDRS‐III, LED, and H&Y data are shown in Figure 1. Median UPDRS‐III scores and LED significantly decreased after 6 months compared to baseline (Figure 1). Wilcoxon signed‐rank test was 0.000 for UPDRS‐III and LED changes and for H&Y change was 0.005. H&Y decreased after 6 months to 2.0 (IQR 2.0–2.5), Wilcoxon signed‐rank test = 0.005. At 6 months, five patients had discontinued levodopa but used dopamine agonists and/or a MAO‐B inhibitor.

At 6 months, 72% of patients had monopolar stimulation on both electrodes, 14% bipolar stimulation and 14% monopolar stimulation on one electrode and bipolar stimulation on another. At 6 months, median stimulation amplitude was 2.3 V (IQR 1.9–2.6 V), pulse width 60 μs (IQR 60), and frequency 130 Hz (IQR 130). Twenty‐six patients (30%) were treated with two different group settings for DBS stimulation, yet 95% of patients had not changed the group settings according to the stored data of IPG. Indications for the different groups were possibilities for the alteration of frequency (82%), pulse width (7%), or electrode contacts (11%).

The median thickness of intracranial air was 3.0 mm on the right and 3.4 mm on the left (IQR 0–5.3 mm on the right and IQR 0–5.9 mm on the left). The midbrain midline shift compared to skull midline measured from preoperative and postoperative CT was preoperatively 0.0 mm (IQR 0.0–1.0 mm) and postoperatively 0.0 mm (IQR 0.0–1.0 mm), Wilcoxon signed‐rank test = 0.83. As the midbrain midline shift from skull midline was similar between pre‐ and postoperative, it was taken to reflect patient anatomy and midline shift was neglected in further analyses.

**Figure 2:**
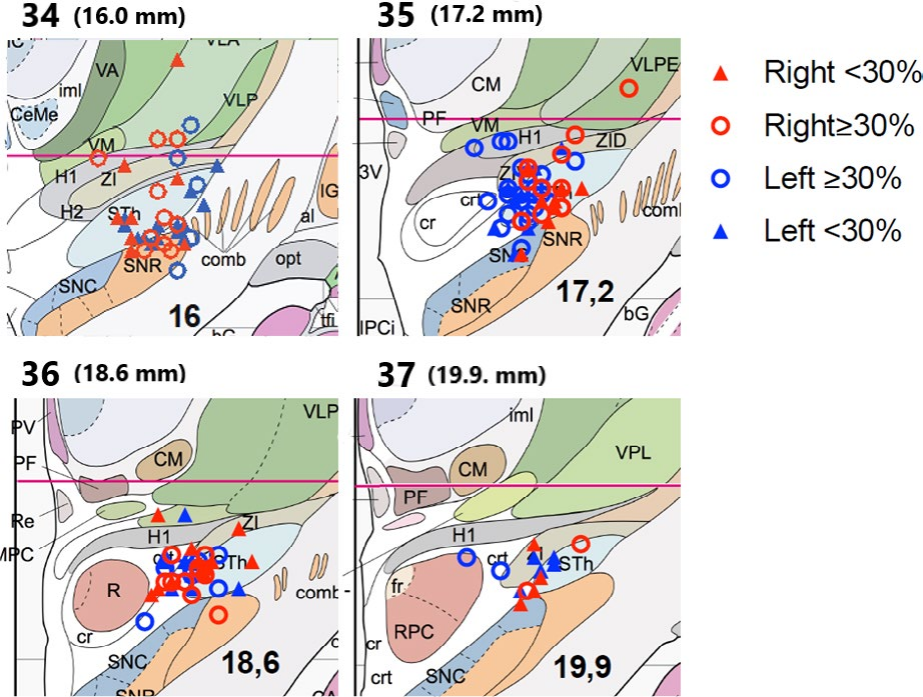
The individual electrodes in reference to Mai Atlas, coronal plates 34–37 in which the majority of electrodes were situated (Mai Atlas plate numbers 34–37 reprinted with the kind permission of Elsevier). In brackets is the distance from anterior commissure to the posterior direction in mm. Abbreviations: al, ansa lenticularis; bG, band of Giacomini; CeMe, central medial thalamic nucleus; CM, centromedian thalamic nucleus; comb, comb system; cr, capsule of red nucleus; crt, cerebello‐rubro‐thalamic fibers; fr, fasciculus retroflexus; H1, thalamic fasciculus (field H1); H2, thalamic fasciculus (field H2); IG, internal globus pallidus; iml, internal medullary lamina of thalamus; IPCi, interpeduncular system; MCP, ventral posterior medial nucleus; parvocellular part; opt, optic tract; PF, parafascicular thalamic nucleus; PV, paraventricular thalamic nucleus; R, red nucleus; Re, reunions thalamic nucleus; RPC, red nucleus; parvocellular part; SNC, substantia nigra; pars compacta; SNR, substantia nigra; pars reticulata; STN, subthalamic nucleus; tfi, tenia of fimbria; VA, ventral anterior thalamic nucleus; VLP, ventral lateral posterior thalamic nucleus; VLPE, ventral lateral posterior thalamic nucleus; external part; VM, ventromedial thalamic nucleus; ZID, zona incerta; 3V, third ventricle

The coordinates calculated with different methods correlated with each other significantly (Spearman correlation coefficient 0.48–0.82, all correlations significant at *p* < 0.001). The median locations and IQR are shown in Table 1 **.** The locations of the individual electrodes in reference to Mai atlas are shown in Figure 2 (Mai et al., 2008).

**Table 1:** Median locations of the electrodes

	Atlas		Nucleus Ruber		MCP	
	Median	IQR	Median	IQR	Median	IQR
Right *X*	11.1	10.3–12.4	3.0	2.3–3.9	12.0	11.1–12.9
Right *Y*	17.2	16.0–18.6	0.1	−1.3 to 1	3.4	2.1–4.3
Right *Z*	4.9	4.1–5.8	1.2	0.0–2.3	3.2	2.0–4.6
Left *X*	10.3	9.1–11.2	1.8	1.0–2.9	10.1	10.1–11.9
Left *Y*	17.2	16.9–18.6	0.1	−0.9 to 1	3.1	1.9–4.3
Left *Z*	4.9	3.7–5.8	1.0	0.0–2.1	3.0	1.5–4.4

Note

Atlas coordinates: *X*, lateral from midline; *Y*, posterior from AC; *Z*, inferior to AC; Nucleus ruber coordinates: *X*, lateral from lateral border of NR; *Y*, posterior from anterior border of NR; *Z*, inferior from superior border of NR; MCP (midcommissural point) coordinates: *X*, lateral from midline; *Y*, posterior from MCP; *Z*, inferior from MCP. All coordinates in millimeters.

The differences of electrode positions in relation to LED reduction are shown in Table 2 in nucleus ruber (NR) coordinates. There was a statistically significant difference in the left dorsoventral electrode location. Patients with greater LED reduction had more ventral *z*‐coordinates in nucleus ruber coordinates (Mann–Whitney *U*‐test = 0.025) and MCP coordinates (Mann–Whitney *U*‐test = 0.007), but not in the atlas‐based coordinates (Mann–Whitney *U*‐test = 0.20). The latter showed only a trend on the right side (Mann–Whitney *U*‐test = 0.10). There was no statistically significant difference of electrode location in patients related to changes of UPDRS‐III at 6 months with the cut‐off of 30% improvement (Mann–Whitney *U*‐test values 0.059–0.975 for the correlations in different coordinate systems).

**Table 2:** Patient groups and electrode coordinates in relation to nucleus ruber (NR) coordinates. Median electrode location corresponds inferomedial part of the STN. STN location in relation to NR as measured from the Mai Atlas is: *X* = 1.9–8.3 mm, *Y* = −3.9–2.7 mm, *Z* = −1.8–1.8 mm

Led decrease at 6 months	In relation to NR	Right			Left		
		*X*	*Y*	*Z*	*X*	*Y*	*Z*
<30%	Median	3.0	0.0	1.1	1.7	0.0	0.6
	*N*	44	44	44	44	44	44
	IQR	2.0–3.9	−1.6 to 0.7	0.0–2.6	2.2–6.6	−1.2 to 1.0	−0.8 to 1.7
≥30%	Median	3.0	0.5	1.4	2.1	0.0	1.6
	*N*	43	43	43	43	43	43
	IQR	2.5–3.8	−0.9 to 1.0	0.4–2.3	1.0–3.0	−0.6 to 1.1	0.3 to 2.3
	Sig.	0.644	0.099	0.131	0.231	0.085	0.025*

*Note*

NR, nucleus ruber; *n*, number of patients; IQR, interquartile range. *Sig < 0.05 statistically significant using Mann–Whitney *U*‐test.

## 4 ADVERSE EVENTS

One intracranial hemorrhage was reported (incidence of 1%), with good recovery. Four ventricle punctures were reported during the operation without clinical significance. Skin infections were the most common infections, either of the IPG (3%) or of the trepanation wound (10%). Fourteen (16%) skin infections were treated with antibiotics, yet only in one patient (1%) the IPG and the electrodes had to be removed due to severe infection. Twenty‐two patients (25%) reported dysarthria related to DBS, which was confirmed by turning DBS stimulation off at DBS programming session. With four patients, the UPDRS‐III subscore number 18 (speech) remained the same at the baseline and at 6 months' follow‐up. Only with four patients (5%), the score increased by 2 points which were regarded as a significant change. With fourteen patients, the score increased by one point.

One patient committed suicide within 6 postoperative months. One postoperative depression was noted and five patients (6%) suffered from a transient confusional state in the first postoperative weeks. No statistically significant difference was seen between electrode contact positions in those patients with neuropsychological adverse effects compared to those without (Mann–Whitney *U*‐test = 0.06–0.98).

## 5 DISCUSSION

The coordinates acquired with different methods correlated significantly with each other. Correlations were under 0.9 in all cases providing support for previous publications that different methods of acquiring coordinates have different validity (Slavin et al., 2006). The previously published studies that have used direct visualization of the STN have been performed with 3 T MRIs (Slavin et al., 2006; Vertinsky et al., 2009). To our knowledge, there are no previous large studies that show good correlation of electrode location acquired by direct visualization of the STN, in relation to nucleus ruber and MCP‐based coordinates from 1.5 T MRIs. MCP‐based coordinates are based on the robust landmarks AC and PC and they are the classical language of stereotaxy, but these landmarks are distant to the STN which might lead to errors due to anatomical variation. The ever‐improving quality of MRI scans is increasing the capability to directly visualize the STN as shown by DBS operation under general anesthesia based in intraoperative MRI (Matias, Frizon, Nagel, Lobel, & Machado, 2018). However, NR is better visualized in MRI than STN and has been found in some studies to more reliable than MCP‐based targeting (Andrade‐Souza et al., 2008; Houshmand et al., 2014; Matias et al., 2018). We suggest that because of anatomical variability and different reliability of the different methods of electrode coordinate acquisition, it may be beneficial to acquire coordinates with at least two different methods. The fact that we found statistically significant differences in coordinates when NR based or MCP coordinates were used seems to support the previous finding that NR‐based coordinates may be a beneficial compromise between reliability MCP coordinate acquisition and validity of direct visualization of STN.

In our study, we found a statistically significant association of dorsoventral contact location with LED reduction only on the left side, that is the more ventral active contact led to greater LED decrease. This novel observation requires more detailed research to be confirmed. Castrioto et al. (2011) have proposed hypothesis of “dominant STN”. In Castrioto's study, the dominancy of STN in 22 patients was determined with bilateral off‐stimulation, bilateral on‐stimulation, unilateral right‐ and left‐stimulation. The cluster analysis of UPDRS‐III scores showed that 11 patients presented with “dominant‐STN.” In Rizzone's study with 10 PD patients with STN DBS, the dominancy of STN could be determined in six patients. Four of these patients had STN dominancy on the right side and two on the left side (Rizzone, Ferrarin, Lanotte, Lopiano, & Carpinella, 2017). Lizarraga, Jagi, & Luca (2016) noted greater improvement of motor symptoms and gait with bilateral stimulation than with unilateral stimulation, yet unilateral right‐sided stimulation had equal effects on gait kinematics but not on the left‐sided stimulation. This STN dominancy phenomenon could have an effect on our observation on dorsoventral contact location with greater LED reduction on left side. The dominancy of STN was not tested on routine DBS programming, so no further analysis could be made.

Hamel et al. conducted a survey to obtain an expert opinion of Parkinson's disease specialists on optimal target for STN stimulation. In this survey, Parkinson's disease specialists were able to point out their preferred position for an active contact in STN on brain atlas. Some experts preferred the dorsolateral STN and subthalamic area, yet the survey concluded that there is no homogenous perception of the optimal anatomical target and the optimal target needs further specification (Hamel et al., 2017). In Garcia‐Garcia's (2016) study (2016), the optimal stimulation site for highest antiparkinsonian advantage was in rostral and most lateral parts of the motor region of STN and at the interface of this region and its adjacent areas (zona incerta and thalamic fasciculus). In Herzog's study with 14 patients, the dorsolateral border of STN or active electrode within STN was most effective when considering motor improvement (Herzog et al., 2004). The possibility of a beneficial effect of a more ventral electrode in our study was found only on the left side where electrodes were also more medial, which correlates with stronger connections to premotor areas, which might explain the finding in part (Romanelli et al., 2004). In addition, STN is located more ventrally in the medial parts. The more ventrally located electrodes might also lead to combined STN and substantia nigra stimulation which has been also studied to provide additional improvement for axial symptoms (Romanelli, Esposito, Schaal, & Heit, 2005; Weiss et al., 2013).

There are some limitations in this study. Firstly, this is a retrospective analysis that restricts interpretation of the results. Secondly, electrode locations analysis is based on postoperative CT scans taken on first or second postoperative day. Kim et al. (2010) demonstrated a significant difference in the electrode positions between the postoperative CT and CT at 6 months. This observation causes a limitation in our study, as the electrode location was determined from postoperative CT scan. However, the observed shift in midline was negligible and the amount of intracranial air was small. Additionally, presentation of median electrode locations between different study groups and the statistical testing of electrode locations may not represent optimal measure of individual treatment outcomes. However, it may be beneficial that DBS studies provide some information of the electrode location. If a suboptimal benefit of STN stimulation is noted postoperatively, an imaging study may reveal exact location of the electrode.

In our study, UPDRS part III scores decreased significantly (40%) during first 6 postoperative months. This is comparable to earlier studies (Deuschl et al., 2006; Weaver et al., 2009). Herzog and Schüpbach have reported up to 51%–53% decrease in UPDRS part III scores (Herzog et al., 2003; Schüpbach et al., 2013). Our results showed a significant LED decrease at 6 months that is in accordance with the previous studies (Herzog et al., 2003; Schüpbach et al., 2013; Weaver et al., 2009). We found less frequent neuropsychiatric adverse effects than previous studies (Herzog et al., 2003), yet the neuropsychiatric side effects occurred during the first postoperative months as described earlier. However, a routine neuropsychological examination at 6 months' follow‐up was not conducted and therefore mild neuropsychological problems might have been unnoticed. There was not any evidence suggesting that those with neuropsychiatric problems had more ventral active electrodes than those with no side effects in our study. This is contrary to observations by Welter et al. (2014) in which the ventral contact location and the form of the disease (younger age, shorter disease duration and higher levodopa responsiveness) related to stimulation‐induced hypomania. This may be due to the methodological differences in studies. Reported rates of ICH were comparable to previous studies (Krack et al., 2003; Weaver et al., 2009).

In this study, a statistically significant correlation between the active contact location and LED reduction was noted. Further research on the correlation between the active contact location, clinical outcome, and LED reduction is warranted.

## DISCLOSURES

Maija Koivu has received a personal grant money from the Finnish Parkinson Foundation and a government grant for academic research in university hospital. Antti Huotarinen has received a personal grant money from the Finnish Parkinson Foundation. Aki Laakso has received a government grant for academic research in university hospital. Eero Pekkonen has received a government grant for academic research in university hospital.
