# Supplementary material for: Motor outcome and electrode location in deep brain stimulation in Parkinson's disease
Source: Brain Behav. 2018 May 30;8(7):e01003. doi: 10.1002/brb3.1003 (PMC6043715; doi:10.1002/brb3.1003)
Supplement: Supplementary file 1 [file BRB3-8-e01003-s001.docx]

**Supplementary Information for the manuscript titled Motor Outcome and Electrode Location in Deep Brain Stimulation in Parkinson’s Disease**


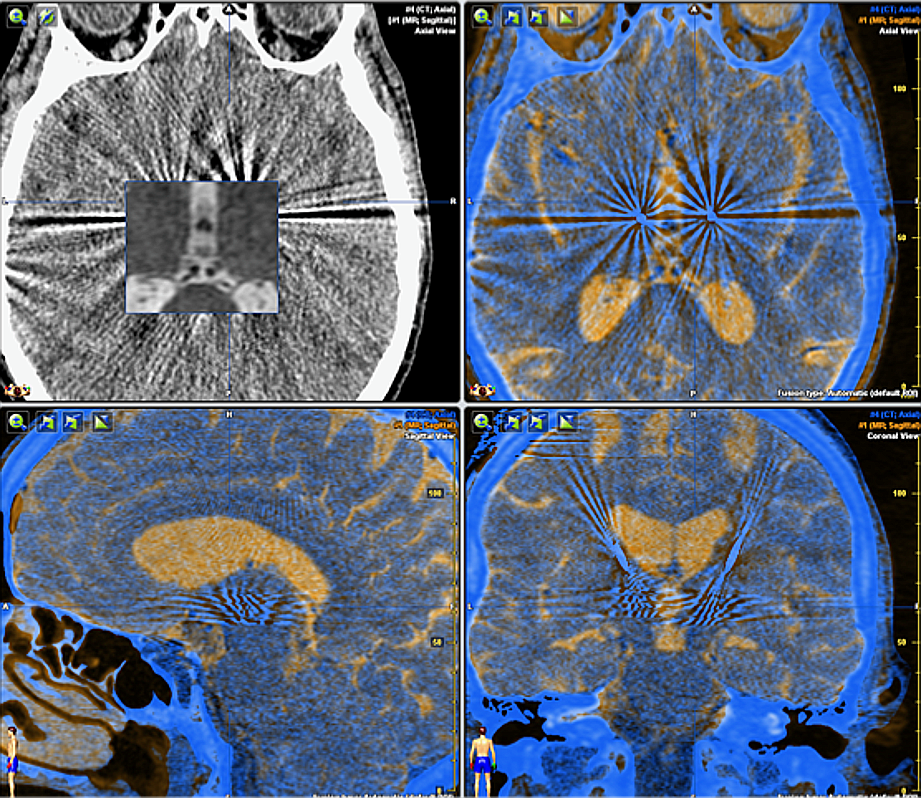


**Supplementary image 1. The fusion of the pre-operative MRI and postoperative head CT.**

**
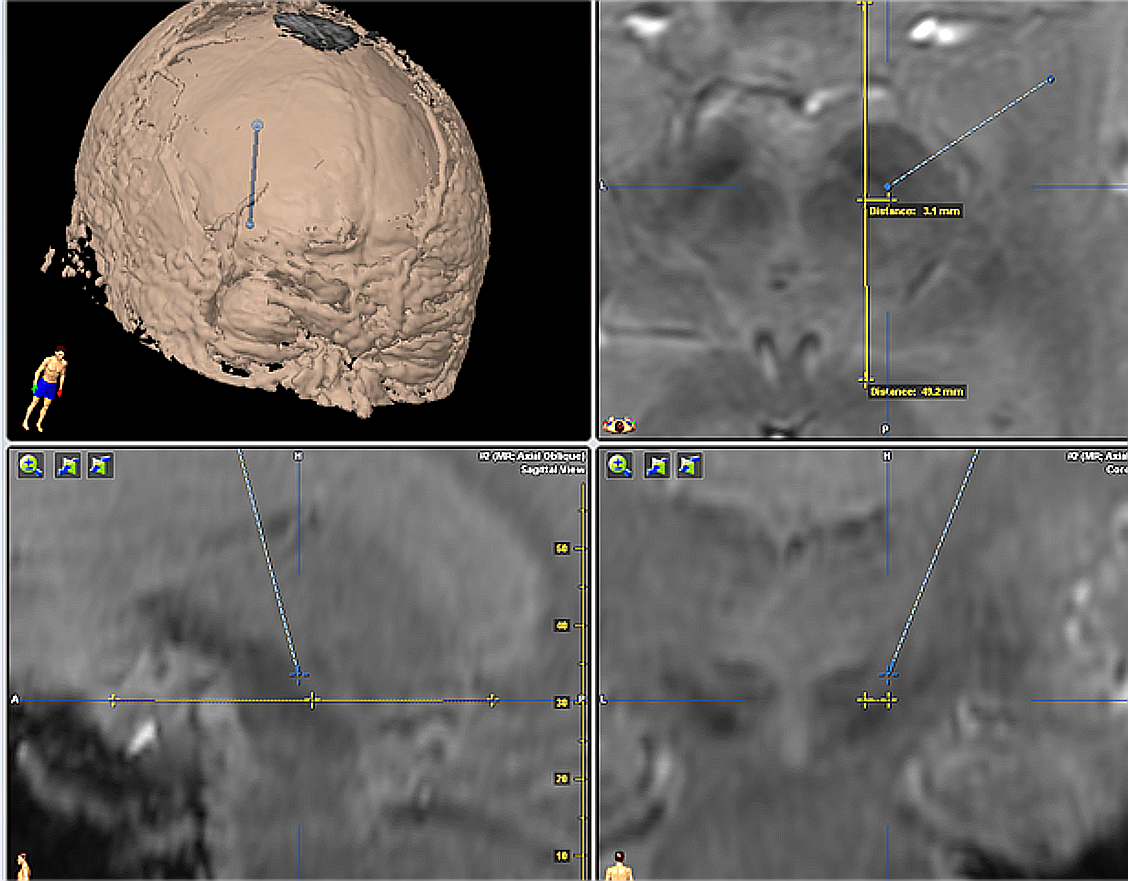
**

**Supplementary image 2. The image of method of measurement of electrode location.**
